# Friction force microscopy of tribochemistry and interfacial ageing for the SiO*_x_*/Si/Au system

**DOI:** 10.3762/bjnano.9.157

**Published:** 2018-06-05

**Authors:** Christiane Petzold, Marcus Koch, Roland Bennewitz

**Affiliations:** 1 INM – Leibniz Institute for New Materials, Campus D2 2, 66123 Saarbrücken, Germany

**Keywords:** contact ageing, friction, nanotribology, tribochemistry, wear

## Abstract

Friction force microscopy was performed with oxidized or gold-coated silicon tips sliding on Au(111) or oxidized Si(100) surfaces in ultrahigh vacuum. We measured very low friction forces compared to adhesion forces and found a modulation of lateral forces reflecting the atomic structure of the surfaces. Holding the force-microscopy tip stationary for some time did not lead to an increase in static friction, i.e., no contact ageing was observed for these pairs of tip and surface. Passivating layers from tip or surface were removed in order to allow for contact ageing through the development of chemical bonds in the static contact. After removal of the passivating layers, tribochemical reactions resulted in strong friction forces and tip wear. Friction, wear, and the re-passivation by oxides are discussed based on results for the temporal development of friction forces, on images of the scanned area after friction force microscopy experiments, and on electron microscopy of the tips.

## Introduction

Contact ageing, the strengthening of contacts after formation, is an important phenomenon in tribology, with impact ranging from the nano-scale (NEMS and MEMS) [1–2] to the macro-scale (sliding of rock in earthquakes) [3–4]. Different microscopic mechanisms for contact ageing have been identified, e.g., material creep [5], structural changes in the interfacial contact [6–7] or an increase in number of chemical bonds [8–9]. The frictional strength may also grow in time by rotation of the contacting surfaces into a preferred misorientation defined by dislocation structure [10], for which friction maxima have been predicted [11].

Friction force microscopy (FFM) is a key method to investigate the microscopic mechanisms underlying friction, wear, and lubrication as it allows for measurements of static and kinetic friction of single nanometer-scale contacts. In FFM, an ultra-sharp tip is scanned across the surface line by line probing a square frame. Lateral forces acting on the sliding contact are determined as deflection of a cantilever spring holding the tip.

Single-asperity contact ageing between silica tip and surface has been directly observed in ambient atmosphere by FFM [8]. It has been explained by an increase in the number of covalent bonds across the contact facilitated by the presence of water [12–13]. Ageing of well-defined contacts between metallic nanoparticles and surfaces has also been quantified by displacement of the particles in a FFM experiment [7].

Contact ageing may proceed at very different timescales for different materials. The initial phase of contact strengthening is often reported to depend logarithmically on the duration of the static contact [5,8]. Cold welding of two Au surfaces in air is a rather slow process (seconds to minutes) [5], while contact ageing between chemically reactive surfaces may occur very fast (nanoseconds to milliseconds) [14].

Here, we report FFM experiments in ultrahigh vacuum that address contact ageing and atomic-scale friction for contacts formed by Si, SiO*_x_*, and Au. We found that no contact ageing was observed for oxidized silicon tips sliding on Au(111) or on oxidized Si(100) surfaces. We therefore deliberately increased the adhesive forces between probes and surfaces. In an attempt to allow for the successive formation of chemical bonds in contact ageing experiments, we removed passivating layers, in particular oxide films, from surface and tip. The control of surface oxidation required the implementation of all experiments in ultrahigh vacuum. We report on tribochemical processes between Si, SiO*_x_*, and Au, which were initiated by removal of the passivating layers.

## Experimental

### Tip and sample preparation

Friction force microscopy experiments were performed with uncoated (“SiO*_x_*/Si”) and gold-coated (“Au/Si”) silicon tips and cantilevers with a nominal spring constant of *k*_nom_ = 0.2 N/m (PPP-CONTR, PPP-CONTAu; Nanosensors, Switzerland). The cantilevers were fixed with conductive glue (H21D; Epoxy Technology, Inc., USA) to a metal tip holder. In order to remove water and physisorbed hydrocarbons, holder and cantilever were transferred into the preparation chamber of an ultrahigh vacuum system (*p* = 10^−10^ mbar) and heated to 120 °C for several hours until the pressure had stabilized. The holder was then transferred into the measurement chamber with the FFM experiment. Normal and lateral spring constant of each cantilever were calculated from the resonance frequency and the dimensions of cantilevers and tips. The tip height was assessed individually from SEM images of the cantilevers before use.

A Au(111) single crystal (MaTeck GmbH, Jülich, Germany) was prepared by repeating a sputter–heating cycle (20 min Ar sputtering at 25 μA/1 keV followed by 1 h annealing at 850 °C) until a sharp (111) pattern was observed by low-energy electron diffraction (LEED). The n-Si(100) sample was cleaved from a wafer and fixed mechanically to a sample holder. The Si was either heated to 120 °C for several hours in the preparation chamber to remove water and physisorbed hydrocarbons (“oxidized Si(100)”), or heated to high temperatures to remove the surface oxide (“reactive Si(100)”). Removal of the oxide layer was achieved by heating with electric current flow through the sample, heating it first to about 600 °C for 8 h (red glow). The current was then increased until the sample was glowing bright orange and no further increase in temperature could be detected when increasing the current (sample temperatures between 1000 and 1300 °C were measured, depending on the sample size and surface oxide). This maximum temperature was kept for about 5 min. The temperature was recorded with a pyrometer (Impac IGA 140; LumaSense Technologies GmbH, Germany). The sample was allowed to cool to room temperature before transferring it to the measurement chamber.

Friction force microscopy experiments were performed with a VT-AFM (Scienta Omicron GmbH, Taunusstein, Germany) under ultrahigh vacuum conditions (*p* < 10^−9^ mbar). While sliding an AFM tip at the end of a soft micro-manufactured cantilever beam perpendicularly to its length axis over the sample surface we recorded the torsional deflection of the cantilever, which allowed for the calculation of frictional forces. The scan rate was kept at 34 ms·line^−1^ if not stated otherwise.

### Slide–hold–slide experiments

Contact ageing between tip and flat surface was investigated in slide–hold–slide experiments similar to those reported in [8], but under UHV conditions at low loads (ca. 0 nN, i.e., the load was controlled by adhesion). As the name “slide–hold–slide” suggests, a tip was slid in contact over a surface, then held stationary for a defined duration of time, and then slid again in the opposite direction. Stationary contact times were varied between nominally 0 and 100 s. In order to realize very short hold times, we further applied different sliding velocities in scanning FFM frames such that the stationary contact duration at the turning points varied between 252 μs and 3.9 ms. Hold times and velocities were varied randomly in order to identify possible systematic errors. The stationary contact in FFM is prone to unwanted movements caused by creep of the piezo actuator and by instrumental drift upon temperature changes. To minimize movement during hold times, the system was allowed to stabilize for prolonged times after turning on the light source for detecting the cantilever bending and after piezo repositioning. Furthermore, the temperature in the laboratory was kept stable to less than ±1 °C. The relative tip movement was monitored during hold periods by recording normal and lateral force signals with an external data recorder (LTT24, Labortechnik Tasler GmbH, Würzburg, Germany). Atomic stick–slip events revealed that the system was drifting by about 1 atom per 10 s in direction of sliding in our experiments.

### Friction force measurements

The friction and wear of five tip–sample material pairs were investigated, namely Au/Si–oxidized Si(100), Au/Si–reactive Si(100), Au/Si–Au(111), SiO*_x_*/Si–reactive Si(100), and SiO*_x_*/Si–Au(111). By activating the tip apex a tribochemical reaction between tip and surface was triggered and the changes in friction were recorded during subsequent scanning. Activating the tip apex in a controlled manner was essential for the experiments. Methods to activate different tips are summarized in Table 1.

**Table 1:** Methods for activating the tip apices. The more controlled method is listed first.

tip	activation

SiO*_x_*/Si	sliding on reactive Si(100); Ar plasma sputtering (10 s) and impacting the surface with a force pulse or an applied voltage pulse
Au/Si	sliding on reactive Si(100); Ar plasma sputtering (10 s) and prolonged static contact on Au(111) for cold welding and subsequent pull-off

Friction force experiments were performed in the following sequence: First, an overview image (1 μm^2^) was recorded with an intact sharp tip. Then a sequence of frames (200 × 200 nm^2^) was scanned in the center of the previous scan area until the friction forces had stabilized. Finally, an overview scan (1 μm^2^) was recorded to reveal topographical changes in the area of the friction sequence. The whole sequence was performed first with tips with intact passivating layer and then repeated with activated tips.

### Electron microscopy (SEM, TEM)

Contamination and modification of the AFM tips were examined by scanning electron microscopy (10 kV acceleration voltage) using secondary-electron and backscattered-electron signals (Quanta 400 FEG; FEI, Eindhoven, Netherlands). The tips were examined before transferring them into the preparation chamber (already glued to the tip holder) for sharpness, tip height, and contaminations, and after friction experiments for changes of the tip shape, material transfer, and tip height.

One Au/Si tip apex was analyzed by transmission electron microscopy (TEM; JEM-2100(HR) LaB_6_, JEOL GmbH, Eching, Germany, 200 kV acceleration voltage) after activation on Au(111) and sliding experiments. The tip was cut using a focused ion beam (Versa3D; FEI, Eindhoven, Netherlands) to a thickness that allowed for TEM analysis. The FIB lamella was produced by first depositing a layer of platinum, starting with an electron beam at 2 kV and subsequently using the Ga ion beam at 30 kV until the platinum layer had reached a thickness of about 2.5 µm. The lamella was cut with the Ga ion beam at 30 kV and 7 nA. Finally, the lamella was cleaned from both sides at 2 kV and 0.26 nA using the Ga ion beam.

## Results

### Atomic-scale friction on oxidized Si(100) and on Au(111)

**Figure 1:**
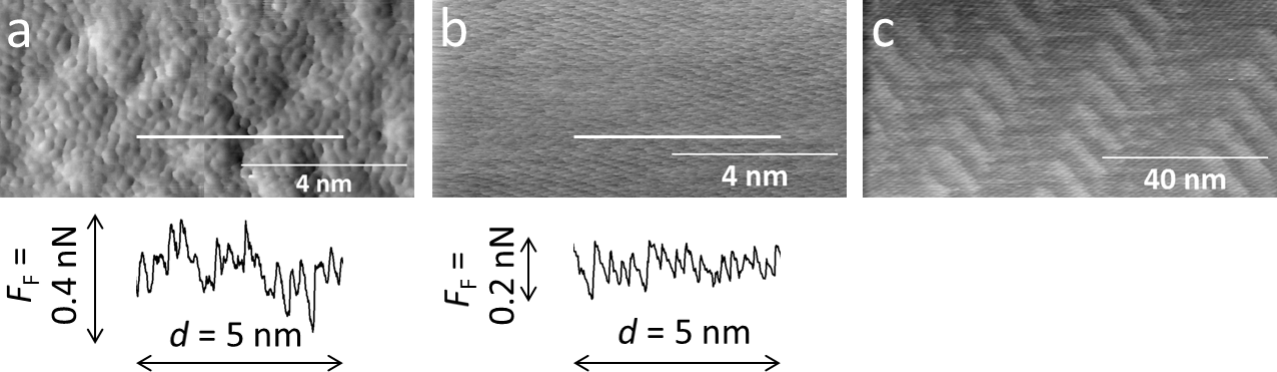
Friction force maps for a) an oxidized Si(100) surface scanned with an intact Ar-sputter cleaned SiO*_x_*/Si tip, b) Au(111), and c) the topography of the Au(111) herringbone reconstruction scanned with an intact SiO*_x_*/Si tip. One line was extracted from a) and b) to visualize the stick–slip pattern on Si(100) and on Au(111).

Sliding an intact Au/Si tip against a non-reactive surface (Au(111) or oxidized Si(100)) typically resulted in friction values in the range of 10–30 pN, while with SiO*_x_*/Si tips the friction was found to be ten times higher (Table 2). Irregular stick–slip signals were detected in the fast scan direction when probing oxidized Si(100) (Figure 1a) and regular stick–slip was detected for the fast scan direction on Au(111) (Figure 1b). For both surfaces a characteristic stick–slip distance in the range of the expected atomic distances (250 pm for Si(100); 170 pm for Au(111)) was observed reproducibly in subsequent scan frames. Larger scan frames recorded on Au(111) sometimes exhibited the herringbone reconstruction (Figure 1c), which is also stable under contact-mode scanning [15].

**Table 2:** Friction values for intact tips sliding against non-reactive surfaces.

tip	surface	

	oxidized Si(100)	Au(111)

SiO*_x_*/Si	0.25 nN	0.33 nN
Au/Si	0.02 nN	0.01 nN

### No contact ageing for passivated tips sliding against non-reactive surfaces

**Figure 2:**
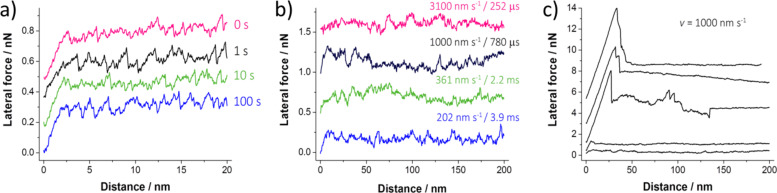
Graphs exemplifying the absence of contact ageing for slide–hold–slide experiments under UHV conditions. All curves are offset for better clarity. a) Variation of the holding times (0–100 s) for a SiO*_x_*/Si tip sliding against a Au(111) surface. The static friction did not exceed the kinetic friction for any holding time. b) Variation of scanning velocity and, thus, of the holding time at the turning points (252 μs to 3.9 ms) for a SiO*_x_*/Si tip sliding against an oxidized Si(100) surface. c) Examples for irregularly large stick–slip events observed during scanning, prevalently at the turning points of the scanning movement. Scan lines are extracted from various positions of two scan frames for a SiO*_x_*/Si tip sliding against an oxidized Si(100) surface at a sliding velocity of 1 μm·s^−1^. The line with the lowest friction corresponds to the black line in Figure 2b. The kinetic friction remained increased after large stick–slip events.

With passivated tips sliding on non-reactive surfaces, we did not observe any static friction peak exceeding the kinetic friction for either of the holding times nor for the turning point at different scan velocities. As examples, we present the lateral force signal of a SiO*_x_*/Si tip sliding against Au(111) for holding times between 0 and 100 s (Figure 2a) and the lateral force signals of a SiO*_x_*/Si tip scanning over an oxidized Si(100) at different velocities (Figure 2b). As we did not observe any static friction peak, overall no contact ageing was found for these non-reactive contacts in vacuum.

Occasionally, we observed first signs of a stronger tip–surface interaction at varying positions and scan velocities, prevalently at the turning points of the scanning motion. The sign for a strong interaction was a sudden increase in friction force where the tip was stuck in contact (Figure 2c). After such isolated large stick–slip events, the kinetic friction remained increased compared to experiments where no friction peaks were observed. Thus, it was not possible to perform systematic slide–hold–slide experiments starting from the same level of friction in each repetition. The stronger interaction occurred at arbitrary positions across the scan frame during repeated scanning, but in most cases the increased stickiness appeared first at the turning points (Figure 2c).

### Tribochemical characteristics of the tip–surface pairings

**Figure 3:**
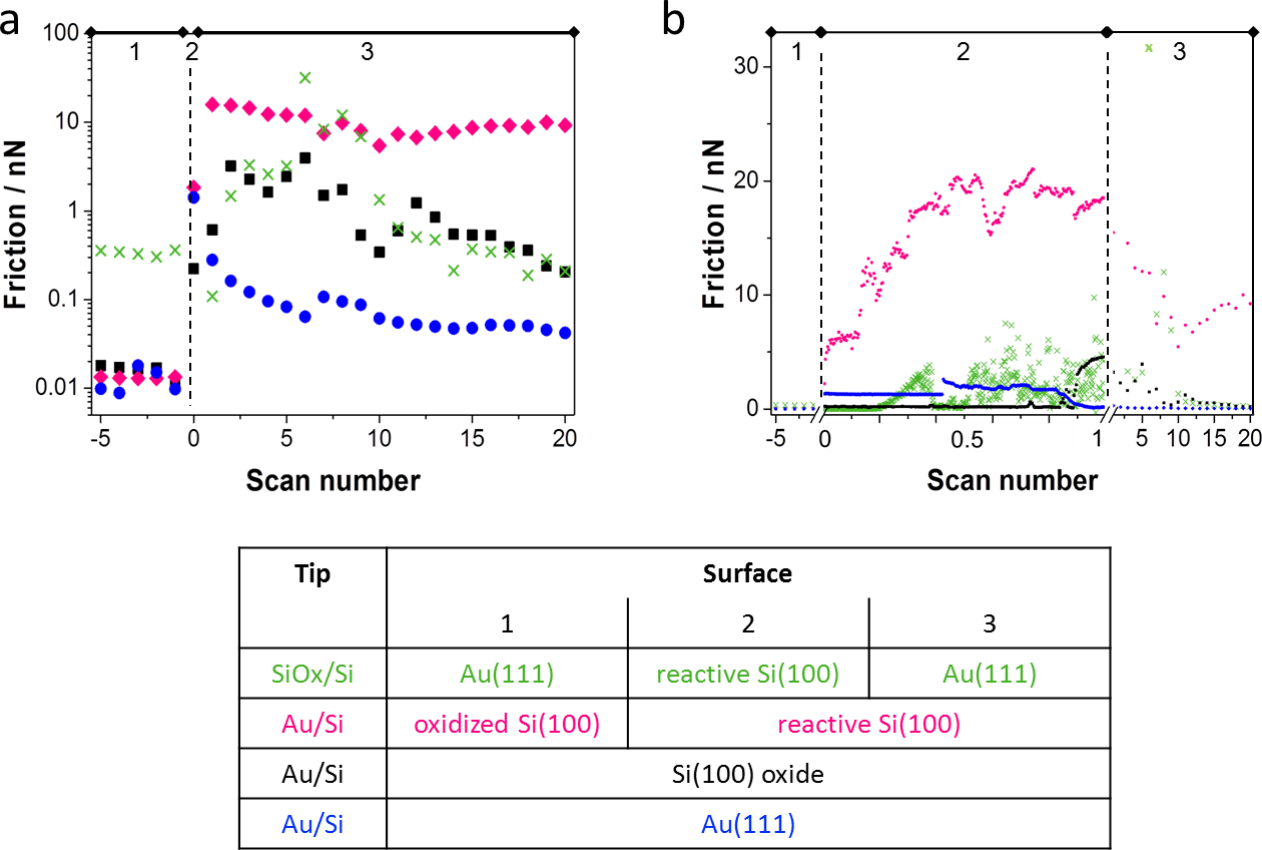
Development of friction for different tip–surface pairs upon activation of the interface. 1 – Control experiment with intact surface and tip; 2 – onset of activation; 3 – friction values for scanning sequences after activation. a) Emphasis on average friction values per scan before and after activation. b) Emphasis on the onset of the interfacial reaction showing the average friction values per scan line for the frame in which the activation occurred.

**Figure 4:**
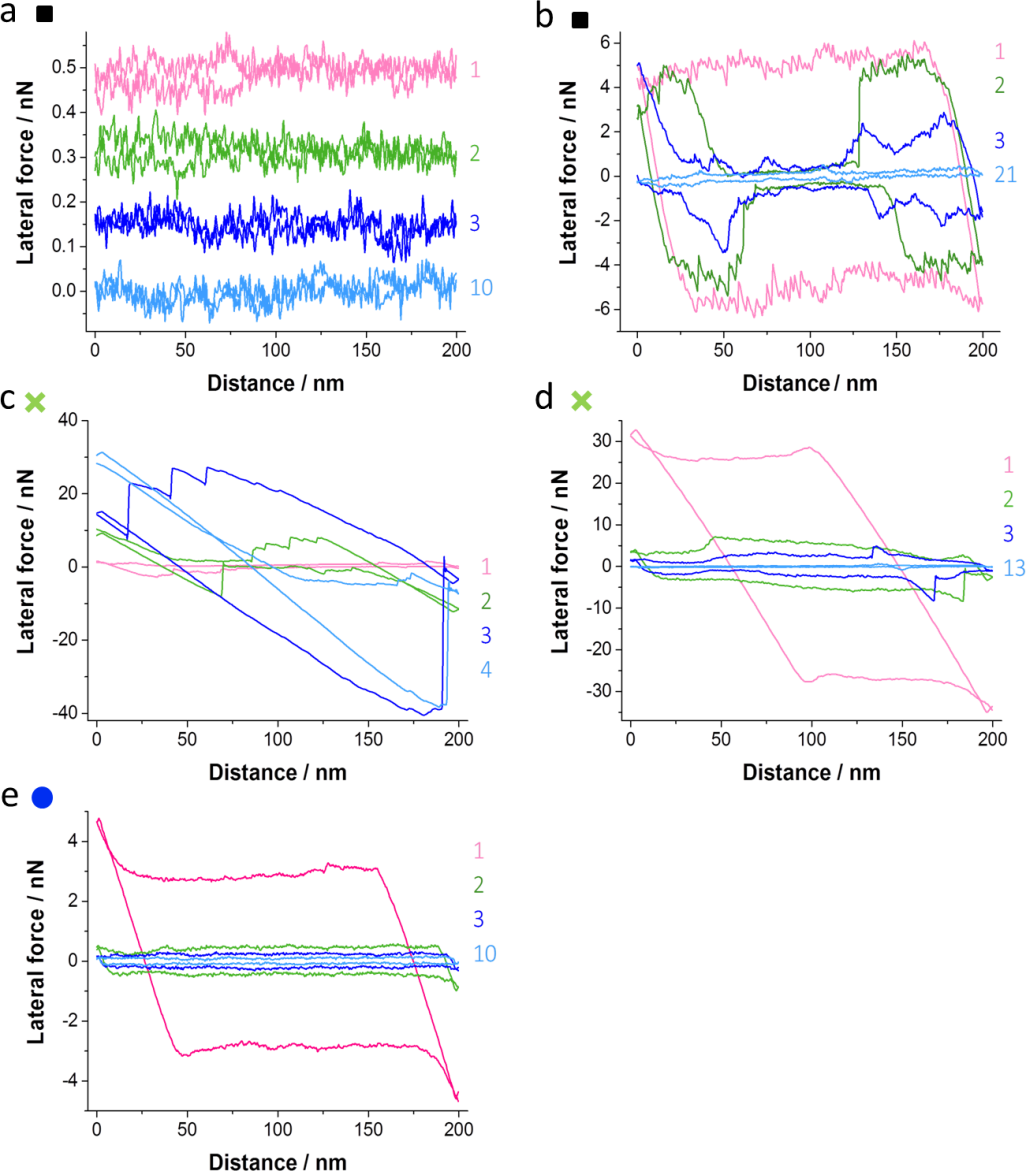
Lateral force loops of the same line from different scan frames. The respective scan number is indicated in the graphs. a) Au/Si tip–oxidized Si(100); friction was very low and decreased with scan frame. The curves are offset for clarity. b) Activated Au/Si tip–oxidized Si(100); the initially high and uniform friction decreased non-uniformly over the surface until it reached a low level and even distribution. c) SiO*_x_*/Si tip–reactive Si(100); the friction increased unevenly with scan frame. d) Activated SiO*_x_*/Si tip–Au(111); the friction decreased from a very high level within 13 scan frames. e) Activated Au/Si tip–Au(111); the friction decreased within ten scan frames and the lateral force remained uniform.

**Figure 5:**
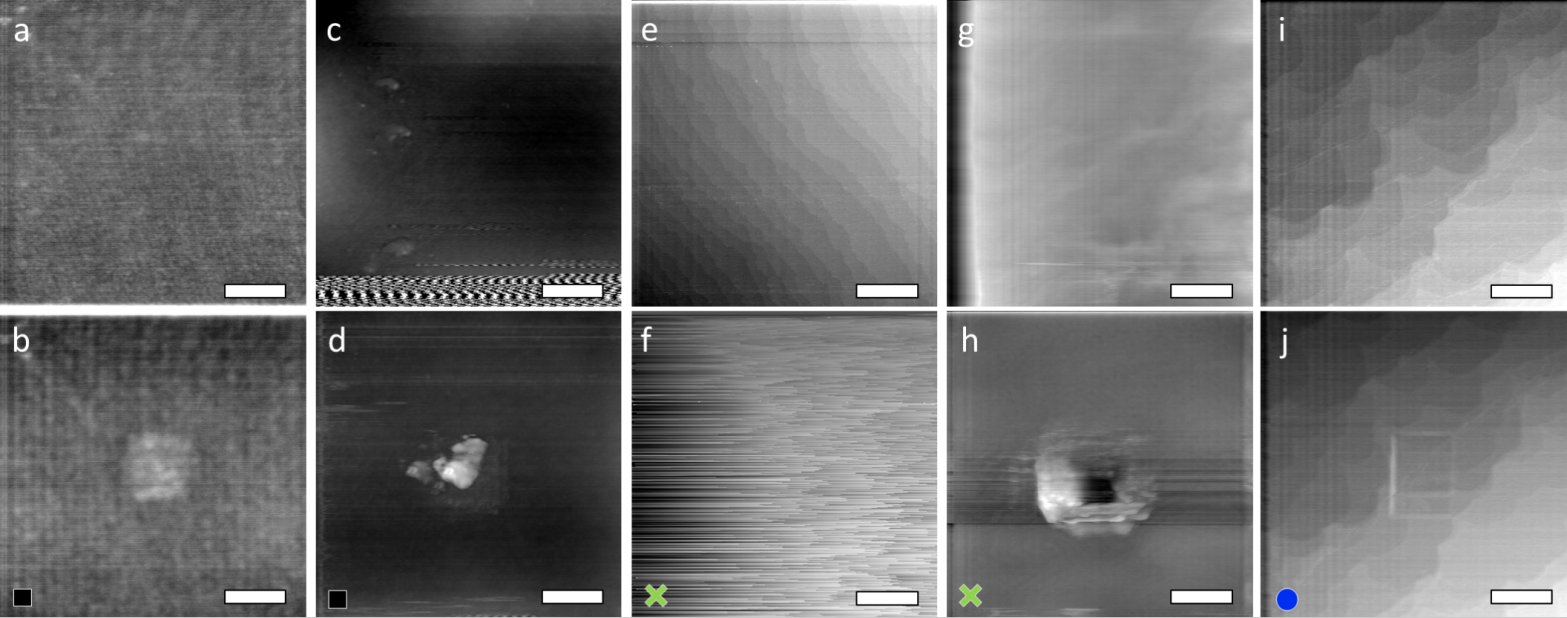
Surface topography of the same area before (upper row) and after (lower row) the friction sequences were recorded in the center of the frames. Scale bars: 200 nm. a, b) Control experiment with an intact Au/Si tip on an oxidized Si(100) surface. Eleven frames were scanned in the center of the scan. The square in the center was elevated by 10 pm. c, d) Activated Au/Si tip on oxidized Si(100). The onset of the tribochemical reaction is revealed in panel c) as intermittent increase in friction causing distortion of the topography measurement. A feature with a height of about 10 nm had evolved in the central area after scanning 21 frames in the image center (panel d)). e, f) Intact SiO*_x_*/Si tip on reactive Si(100). Monatomic steps were observed in panel e); after scanning four frames the topography measurement became hampered by high adhesive friction (panel f)). g, h) Activated SiO*_x_*/Si tip on Au (111). After 15 scans in the center, the topography had changed significantly. The height of the pile-up around the square depression is about 5.8 nm (panel h)). i, j) Intact Au/Si tip sliding on Au(111); despite low normal forces applied and low friction force measured, scanning ten frames in the center resulted in topographical changes. The scanning area had been offset after five scans; therefore two overlapping squares can be distinguished. The height of the pile-up at the left edge of the squares is about 1.15 nm (panel j)).

**Figure 6:**
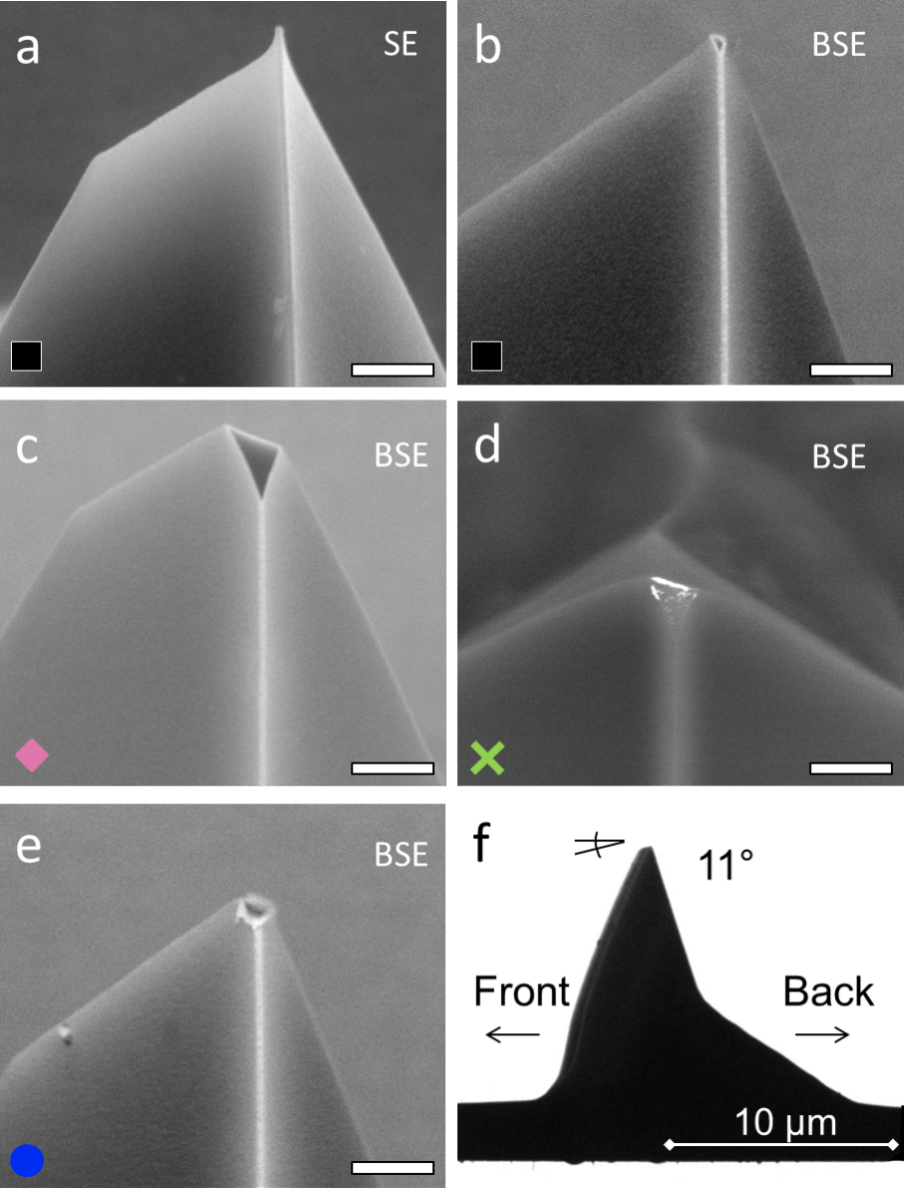
Scanning electron microscopy (SEM) images (secondary electrons (SE), back-scattered electrons (BSE)) of tip apices used in the experiments. The scale bars in panels a) to e) correspond to 500 nm. a) Au/Si tip after sliding for a distance of 4.8 mm on oxidized Si(100), with no detectable wear. b) Au/Si tip after activation and sliding for a distance of 20 mm on oxidized Si(100); the tip was worn by 325 nm. c) Au/Si tip after sliding for a distance of 10 mm on reactive Si(100); the height difference to the intact tip was about 530 nm. d) SiO*_x_*/Si tip after sliding for a distance of 17.7 mm on reactive Si(100) and Au(111); the tip was worn by 880 nm and Au was transferred from the surface to the tip apex (white contrast). e) Au/Si tip after activation and sliding for a distance of 260 mm on Au(111); tip wear was 370 nm. f) TEM side view of a non-coated AFM tip after sliding. The angle of the worn tip apex corresponds to the angle between cantilever holder and surface of 11°.

We suggest that the intermittent friction peaks (Figure 2c) were caused by chemical bonding between tip and surface after local damage of a passivating layer on tip and/or surface. To clarify the origin of the large stick–slip events and of the increase of kinetic friction we removed the protective oxide layer from either the tip or the surface and created a tribochemical system of Au, Si, and SiO*_x_* (Table 1). Different aspects of tip–surface interactions are illustrated by the average friction forces (Figure 3), single lateral force traces from the scanning sequences (Figure 4), surface topography images recorded before and after the sliding sequences (Figure 5), and electron microscopy images of the tips before and after the experiments (Figure 6).

In general, the onset of tip activation was evident by a sudden increase in friction (Figure 3a,b). Results for the tip–surface interactions of the different tips and surfaces are presented in this section. Friction of a Au/Si tip sliding on oxidized Si(100) is illustrated by the black squares in Figure 3. Initially (section 1 in Figure 3) a low friction force of about 0.02 nN was measured for the intact tip sliding against the intact oxide surface. Friction decreased slightly with scanning time until stabilizing after about 10 scans (Figure 4a). We attribute this decrease to structural changes at the sliding interface caused by tip–surface interactions, as scanning the same area ten times left an elevation of about 10 pm in height (Figure 5a,b). This elevation is less than the diameter of Si (111 pm) and Au atoms (144 pm). We thus exclude material transfer and rather assume structural changes at the atomic scale. We did not observe any shape changes at the tip apex by SEM analysis after a total sliding distance of about 4.8 mm (Figure 6a).

The same Au/Si tip was activated by Ar sputter cleaning and impacting on the surface of oxidized Si(100) (Table 1). Continued sliding on oxidized Si(100) in a different area then resulted in a sudden increase in friction towards the end of the first scan frame (section 2 in Figure 3b, Figure 5c), where friction forces increased to about 3 nN. Friction decreased during the following scans (section 3 in Figure 3). This decrease of friction proceeded by localized changes of the surface. It started from a circular area of decreased friction in the scan center that became wider until the friction was reduced to about 0.2 nN in the entire scan area after 20 scans (Figure 4b). An overview scan of the area revealed material transfer to the oxidized Si(100) surface (Figure 5c,d) with an elevation of about 10 nm. The tip apex was worn by about 325 nm after a sliding length of 10 mm after tip activation (Figure 6b). We did not observe material transfer to the tip apex by electron microscopy.

The initial friction forces measured for another non-activated Au/Si tip sliding against oxidized Si(100) (pink diamonds in Figure 3) were again below 20 pN. Changing the sample and sliding this Au/Si tip against reactive Si(100) resulted in a very rapid increase of friction forces (section 2 in Figure 3b) to more than 20 nN. Friction remained at this level throughout the scanning sequence (section 3 in Figure 3). The tip apex was worn flat by 530 nm after a total of 10 mm sliding distance with no material transferred to the tip (Figure 6c). Observing changes of the topography of the reactive Si(100) surface was not possible as the tip adhered strongly to the surface, leading to artifacts in the topography measurement (similar to Figure 5f).

The initial friction between a SiO*_x_*/Si tip and an oxidized Si(100) surface (green crosses in section 1 in Figure 3) was about ten times higher than the friction between Au/Si tips and oxidized Si(100) (black and pink symbols). Interestingly, the onset of the tribochemical reaction between this SiO*_x_*/Si tip and a reactive Si(100) surface (green crosses in section 2 of Figure 3) was later and the increase in friction slower compared to the Au/Si tip on reactive Si(100) (pink diamonds in section 2, Figure 3b). It was possible to scan one entire frame on reactive Si(100) before friction increased gradually during the second scan to about 5 nN and later to more than 10 nN (Figure 4c, Figure 5e,f). The maximum friction after four scan frames was still lower than for a Au/Si tip on reactive Si(100). Again, the friction did not decrease for continued sliding on reactive Si(100) (results not shown). This activated SiO*_x_*/Si tip was subsequently used for a sliding experiment against Au(111). After an initial increase of friction forces to 32 nN, we found a rapid decrease to a level comparable to that of Au/Si tips sliding on oxidized Si(100) (Figure 3, green crosses in segment 3; Figure 4d). The scanning produced a square hole with material shifted to its borders (Figure 5g,h). Electron microscopy of the tip apex revealed material transfer from the Au(111) surface to the tip (Figure 6d). The tip was worn by 880 nm after a sliding distance of 17.7 mm.

Scanning a Au/Si tip against the Au(111) surface at low loads resulted in low friction of about 0.01 nN (blue dots in Figure 3a, section 1) and modification of surface topography by displacement of surface atoms (Figure 5i,j). To compare tribochemical reactions of SiO*_x_*/Si and Au/Si tips with the Au(111) surface, a sputter-cleaned Au/Si tip was activated by leaving it in stationary contact for 48 h until cold welding had occurred and adhesive forces between tip and surface had become larger than the force detection range of the cantilever. We found that the tip was reactive after pulling it off the surface, indicating a partial removal of the Au coating. The friction force was increased to 1.4 nN followed by an increase to about 3 nN while scanning the first frame (blue circles in section 2, Figure 3). In the following sequence of scan frames, friction decreased faster and to lower values than for the activated tips described above (section 2 in Figure 3b, Figure 4e). After a total sliding distance of 260 mm the tip apex was worn by about 370 nm. The electron microscopy images of the Au/Si tip slid against Au(111) show a rim of gold with an irregularly shaped border (Figure 6e) rather than a very sharp border as in the tips worn flat against Si(100) oxide and reactive Si(100) (Figure 6b and Figure 6c, respectively).

The lateral force loops in Figure 4 provide additional details on how friction developed for the tip–surface pairings. The friction force *F*_F_ (also given in Figure 3) is calculated from the lateral forces *F*_L_ measured in forward (FW) and backward (BW) direction as 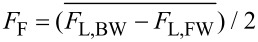, where the average of lateral forces is taken over each scan line. Figure 4 presents the same scan line from subsequent scan frames. The lateral force loops for the Au/Si tip sliding on oxidized Si(100) (Figure 4a) show a very low initial friction (2 pN) and even a decrease of friction from scan 1 to scan 10 to a friction value of 0.2 pN. When the same tip was activated (Figure 4b), friction first increased to about 5 nN and then decreased in the course of the scanning sequence to 0.1 nN in a non-uniform manner. Patches of low friction grew from one scan frame to the next, indicating the formation of a low-friction layer with weak an irregular stick–slip pattern on the surface. When sliding a SiO*_x_*/Si tip against reactive Si(100), the friction increased with sliding time (Figure 4c). Large stick–slip events extended across almost the entire scanning distance (scans 3 and 4 in Figure 4c) due the strong tip–sample adhesion. Sliding activated tips against Au(111) always resulted in a decrease of friction, both for activated SiO*_x_*/Si tips (Figure 4d) and activated Au/Si tips (Figure 4e).

Additional information about the friction processes can be obtained by comparing topography features before and after the sliding sequences (Figure 5). An intact Au/Si tip left an elevated square with a height of only some picometers on oxidized Si(100) (Figure 5a,b), but on Au(111) it moved Au atoms to the four sides of the scan frame (Figure 5i,j). From an activated Au/Si tip material was transferred to the oxidized Si(100) surface (Figure 5c,d) up to a height of 10 nm, while an activated SiO*_x_*/Si tip created a square depression with large pile-up on all sides on the Au(111) surface (Figure 5g,h).

Electron microscopy of the tips after sliding on the various surfaces reveals distinct differences in shape and material transfer for different tip–surface pairings (Figure 6). No changes at the tip apex were observed after sliding an intact Au/Si tip on oxidized Si(100) for 4.8 mm (Figure 6a). A tip wear of 325 nm was measured when the same tip was activated before sliding for 20 mm on oxidized Si(100) (Figure 6b). Prolonged stationary contact with Au(111) after sputtering resulted in activation of a Au/Si tip. After sliding this tip for 260 mm on Au(111), the tip was worn by 370 nm (Figure 6e). The largest wear was measured for tips that had been sliding against reactive Si(100); the Au/Si tip was worn by 530 nm after a sliding length of 10 mm (Figure 6c), and the SiO*_x_*/Si tip was worn by 880 nm after a sliding length of 17.7 mm (Figure 6d). A wear rate was determined by estimating the wear volume as that of a cone with the base area equal to the flat area of the worn tip, with the height of the measured height loss, and the apex radius of the un-worn tip. The wear rates are summarized in Table 3 for the cases described above. We refrain from including the normal force into the calculation of the wear rate since the unknown contribution of adhesion is significant in these nanoscale experiments. The highest wear rate was found for the tips that had been in contact with reactive Si(100). Material transfer was found in one case, for the activated SiO*_x_*/Si tip after sliding on Au(111) (Figure 6d). The coatings of the Au/Si tips that were slid against reactive or oxidized Si(100) had a sharp border (Figure 6b,c), while the Au coating of the Au/Si tip that was slid against Au(111) had an irregularly shaped border (Figure 6e). When viewed from the side, the wear of the tip apices corresponded to the sliding angle of about 11° between cantilever and surface (Figure 6f).

**Table 3:** Wear rate estimated from SEM tip images. Please refer to the description in the text for details on the surfaces and the tip activation, for example by intermittent sliding on reactive Si(100).

tip	surface	*V*_worn_ / 10^6^ nm^3^	*d* / mm	wear rate / 10^5^ nm^3^·mm^−1^

Au/Si (black squares)	Si(100) oxide	3.5	20	1.7
Au/Si (purple diamonds)	Si(100)	15.7	10	15.7
SiO*_x_*/Si (green crosses)	Au(111)	66.3	17.7	37.5
Au/Si (blue circles)	Au(111)	6.1	260	0.2

### TEM analysis of the damaged Au/Si tip after sliding on Au(111)

**Figure 7:**
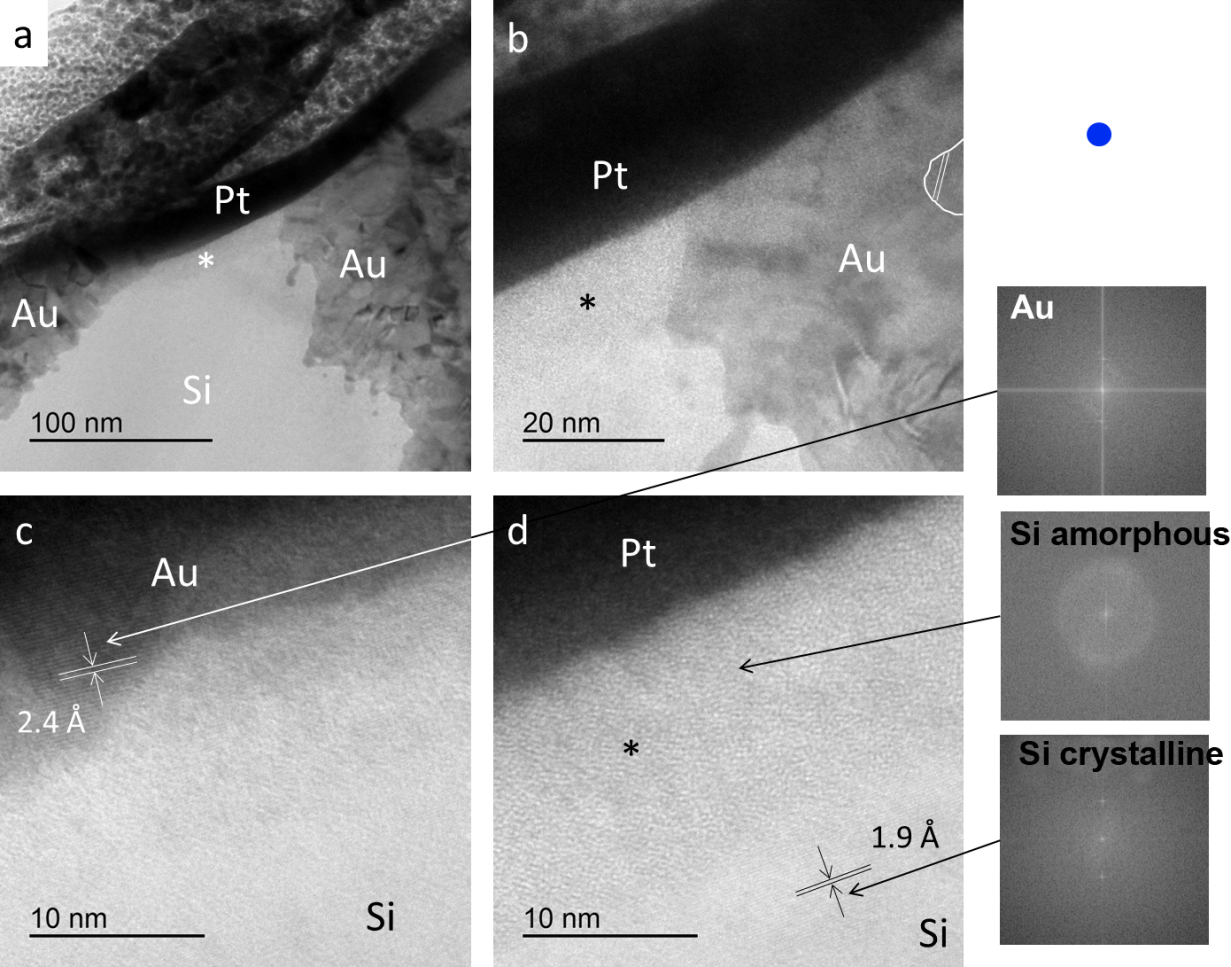
TEM images of a cross section through the apex of a Au/Si tip that had been sliding against Au(111). a) Overview showing the Pt protective coating that was added prior to FIB cutting, the Au cantilever coating and the Si bulk. The contact area is worn flat and the asterisk marks an amorphous layer in the contact region. b) Detail of panel a) showing the amorphous Si with its sharp boundary to crystalline Si as well as crystallites (highlighted) in the Au layer. c) Close-up resolving the crystal lattice in the Au coating layer. The lattice distance was measured to be 2.4 Å. d) Detail of the amorphous layer (*) and the crystalline Si bulk with a lattice distance of 1.9 Å. Fourier transformations of the Au lattice in panel c) and the Si amorphous layer and the crystalline Si from panel d) are provided in the right part of the figure.

The cross section of a Au/Si tip that had been sliding against Au(111) (Figure 6e) was analyzed by transmission electron microscopy (TEM). The Au coating and the Si bulk can be clearly distinguished in the images (Figure 7a,b). In some areas a crystalline lattice was resolved (lattice spacing of 2.4 Å in the Au layer and 1.9 Å in the Si bulk are indicated in Figure 7c,d). The Au coating consisted of crystal grains of different orientation. Tip wear proceeded through the Au coating and the SiO*_x_* layer (Figure 7a). Low amounts of Cr were detected by EDX analysis, especially in the Au layer, while no adhesive Cr layer could be distinguished between Si and Au. An amorphous layer (marked with asterisk in Figure 7) with a thickness of about 13 nm covers the surface that had been in sliding contact with Au(111). A rather sharp border marks the transition from amorphous Si to crystalline Si (Figure 7d). If the amorphous layer contained significant amounts of oxygen, it would appear brighter than the crystalline silicon. This was not the case and we conclude that the amorphization is a mechanical process that does not require oxidation to occur. Fourier transformation of the images (right side of Figure 7) revealed crystalline order for the tip material lattice and amorphous short-range structure for the layer that was transformed by the sliding contact.

## Discussion

We start by discussing the absence of contact ageing in our slide–hold–slide experiments by AFM in ultrahigh vacuum. Previous experimental results had suggested the formation of a liquid-like Au neck in nanometer-scale single-asperity contacts on gold surfaces by diffusion of Au atoms along surface and contact [15–19]. We assumed that the neck formation by diffusion would manifest itself as contact ageing in slide–hold–slide experiments on the time scale of seconds, similar to the development of grain boundaries in the fusion of gold nanoparticles [16]. In our experiments, the holding time before sliding was varied between 252 μs (*v* = 3.1 μm·s^−1^) and 100 s. As our system was drifting by only one atomic distance per 10 s, we should be able to detect effects of the holding times up to 10 s. No static friction peak was observed for any of the holding times for SiO*_x_*/Si tips sliding against Au(111). Au atoms are very mobile on Au(111) surfaces; at room temperature a Au atom may diffuse several micrometers within nanoseconds [20]. We conclude that contact ageing on Au(111) that results from Au atom diffusion is too fast to be observed with our experiment, or that the neck formation by Au diffusion does not result in increased static friction as the neck follows the AFM tip movement without additional friction. In this case we may even ask if the neck actually follows the tip movement or, considering the fast diffusion rates of Au, if it is rather re-forming continuously in a liquid-like manner.

We also investigated contact ageing for SiO*_x_*/Si tips on oxidized Si(100) surfaces, a system for which contact ageing in slide–hold–slide experiments in ambient atmosphere has been reported [8]. While we could reproduce the result in ambient atmosphere, we did not observe any static friction peak in ultrahigh vacuum for holding times up to 100 s. The absence of contact ageing in vacuum is in agreement with the explanation that contact ageing in air is caused by a successive formation of hydroxide bonds between the oxide surfaces, which requires the presence of water [12–13,21].

When probing Au(111) with an intact, passivated tip we found regular stick–slip patterns in the lateral force signal, which resembled the atomic structure of the (111) surface. Despite the small friction forces in the piconewton range, scanning resulted in pile-up of atoms at the edges of the scan frame. It is striking that the observation of atomic stick–slip friction does not imply sliding on a perfect rigid atomic lattice but rather a tip interacting with a dynamic surface of displaceable atoms. This description is in full agreement with the explanation of the absence of contact ageing by a liquid-like nature of the contact. An irregular atomic-scale stick–slip pattern was observed for the oxidized Si(100) surface. The irregularities were likely caused by the nanoscale topography of the oxide surface. Patterns with larger characteristic slip length and signs of stronger tip–surface interactions were recently reported for similar tip–surface pairings at low pressure [22]. The difference between the observations could be caused by the difference in water and oxygen content between experiments in low vacuum and in ultrahigh vacuum with extended surface cleaning, as water and oxygen cause the formation of hydroxide bonds between two SiO*_x_* surfaces [8,12].

Since we did not observe contact ageing for the intact tips, we developed several techniques for controlled removal of passivating layers from the tip apex or the counter surfaces. The onset of chemical interactions between tip and surface was very sudden and resulted in a strong increase in friction forces. Significant static friction peaks appeared, mainly at the turning points of the scanning motion but also at random positions in the scan frames. Once the tip had been stuck to a position on the surface and a significant static friction peak had been observed, the kinetic friction remained increased during the subsequent sliding compared to experiments where no static friction peaks were encountered. The static friction peaks varied in magnitude and could not be reproduced by repeating the scan at the same position. We conclude that sticking of the contact and breaking loose changed the local surface chemistry as well as structure and chemistry of the tip. AFM-based slide–hold–slide experiments require that structure and chemistry of the tip–surface interface are similar at the beginning of each holding phase [7–8]. This prerequisite could not be met by our AFM experiments on reactive SiO*_x_*/Si and SiO*_x_*/Si/Au systems in ultrahigh vacuum.

The onset of reactivity between tip and surface occurred faster when sliding Au/Si tips on reactive Si(100) than for SiO*_x_*/Si tips. The difference can be explained by the thickness of the oxide layer on the tip, which is thinner for Au coated tips than for the oxide-sharpened SiO*_x_*/Si tips. Furthermore, the highly reactive Cr that is used in Au-coated tips as thin adhesive layer could play a role in the chemical activation and act as a catalyst.

Strong friction was observed as sign of reactive tip–sample interactions after removal of passivating layers. The further development of friction after tip activation depended crucially on the presence of atoms that could re-passivate the interface. The effects of passivation have been shown in pioneering study on UHV AFM by Howald et al., who achieved stable sliding on reactive Si(111) after covering the tip by PTFE [23]. For our tips, sliding contacts with clean Si(100) stayed reactive and were subject to adhesive wear, as impressively shown by the SEM images of the worn tips. The removal of the oxide layer and later of the Si from the sliding tip can possibly be enhanced by the formation of volatile silane with residual H_2_ molecules in the UHV chamber and with H atoms passivating Si(100), as we did not find any wear debris on the tips or on the surfaces. Wear rates of sharp AFM tips after sliding distances of up to 1 μm against diamond have been calculated earlier [24]. The wear volumes and sliding distances were much smaller than in our experiments but the wear rates of (5–100) × 10^5^ nm^3^·mm^−1^ are comparable with the present results. The initial wear rates in [24] are expected to decrease with sliding distance due to reduced pressure at the interface and cannot be extrapolated linearly [25].

When two reactive surfaces were in sliding contact we measured very high lateral forces. The AFM tips were worn flat with an angle imposed by the geometry of the AFM. The flat was smooth and no wear debris was found. Therefore an adhesive wear process where single atoms were removed through formation and breaking of chemical bonds is a most likely microscopic scenario [24–25].

A reactive contact sliding on oxidized Si(100) or Au(111) will be passivated within a few scan frames. As the effective contact time for each position while scanning was in the range of microseconds, the passivating reaction occurred on this fast time scale. Intermediary formation of gold silicide at the sliding interface is expected only at higher temperature [26–27] but may be facilitated by frictional energy and by shear mixing. Eutectic AuSi will segregate at room temperature and can thus not be detected after the experiments. We rather suggest that for re-passivation Si reacts with oxygen-containing species on oxidized Si(100) and Au(111) surfaces.

Higher friction forces were measured after activation and re-passivation compared to the initial values. They were likely caused by the increased contact area [28] and the modified geometry of the tip, the flat end of which was worn to be perfectly parallel with the surface. The results of Figure 3 and Figure 4 reveal details on the transfer of material in the scanning process. Both on oxidized Si(100) and on Au(111), scanning with passivated, intact tips led to very subtle atomic-scale rearrangements on the surface despite minimal friction forces. Neither the atomic roughening of the oxidized Si(100) nor the displacement of gold atoms to the edges of the scanning frame on Au(111) caused a significant contact ageing. While there was significant loss of material from the tip due to wear, no corresponding volume was found on the surfaces when imaging after the scanning sequences. Transfer of surface material to the tip was observed only for gold and after scanning on Au(111) surfaces. The transferred gold was spread across the worn contact for SiO*_x_*/Si tips and stuck to the Au coating in the case of Au/Si tips.

Amorphization of the Si tip apex after sliding contact with silicon oxide in air and vacuum has been reported before [29–31] and was confirmed here for sliding contacts in UHV by TEM analysis of the tip. The thickness of the amorphous layer on our tip was in the same range as described for scratching experiments on oxidized Si [29–31]. Upon scratching in air, the amorphization is accompanied by changes in chemical composition towards a higher oxide content [30] and by distortions of the crystalline structure below the scratch [29]. Our TEM images indicate that the friction-induced amorphization proceeds with similar results for silicon in UHV, probably without significant oxidation. Precise determination of the chemical composition in nanometer-sized regions of the tip apex lamella by EDX or from scattering results in TEM remains a challenge, in particular as the sample is exposed to air during preparation and transfer.

## Conclusion

In summary, we investigated nanometer-scale friction phenomena between oxidized and clean silicon surfaces and gold surfaces by means of friction force microscopy in ultrahigh vacuum. We did not observe any contact ageing for oxidized silicon tips sliding on gold surfaces or on oxidized silicon surfaces, although we found evidence for atomic rearrangement in the course of scanning. We conclude that the contact formation with the gold surface is too fast and the contact too compliant to reveal contact ageing as holding-time-dependent static friction. For friction between oxidized silicon surfaces, the chemical bond formation leading to contact ageing does not occur in the absence of water in UHV. Both systems show very low lateral forces compared to pull-off forces, modulated by the atomic structure of the surface.

When passivating layers are removed from tips or surfaces, tribochemical reactions cause a strong increase in friction and significant tip wear in the Si/SiO*_x_*/Au system. On reactive, clean Si(100) we found persistent adhesive wear, while the presence of oxidized silicon led to a re-passivation of the sliding interface within a few scan frames.
